# Resveratrol Inhibits CD4^+^ T Cell Activation by Enhancing the Expression and Activity of Sirt1

**DOI:** 10.1371/journal.pone.0075139

**Published:** 2013-09-20

**Authors:** Ting Zou, Yi Yang, Fei Xia, Anfei Huang, Xiaoming Gao, Deyu Fang, Sidong Xiong, Jinping Zhang

**Affiliations:** 1 Institutes of Biology and Medical Sciences, Soochow University, Suzhou, Jiangsu Province, People’s Republic of China; 2 Department of Pathology, Northwestern University, Feinberg School of Medicine, Chicago, Illinois, United States of America; Institut national de la santé et de la recherche médicale - Institut Cochin, France

## Abstract

Resveratrol, a natural polyphenol compound, has broad effects on critical events, including inflammation, oxidation, cancer and aging. However, the function and molecular mechanisms of resveratrol on T cell activation are controversial. In the present study, we found that resveratrol significantly inhibits the activation and cytokine production of T cells in vitro and in vivo. Sirt1 expression was up-regulated in resveratrol-treated T cells. Once Sirt1 was down-regulated in the T cells, the resveratrol-induced inhibition of T cell activation noticeably diminished. The acetylation of c-Jun decreased and its translocation was impeded in the resveratrol-treated T cells. The incidence and severity of collagen-induced arthritis in the resveratrol-treated mice were considerably reduced.

## Introduction

Resveratrol is a natural polyphenol compound found in rich quantities in mulberries, peanuts, grapes, and red wine [1,2]. Resveratrol has elicited much research interest following the discovery of its cardioprotective properties, which have been associated with drinking red wine. This association has been used to partially explain the “French paradox”, which is the observation of a low incidence of coronary heart disease in France [3]. Since then, several studies have reported the effects of resveratrol on critical events, including inflammation, oxidation, cancer and aging [4–8].

Some studies have shown that resveratrol also affects the immune response. Gautam et al. were the first to investigate the effects of resveratrol on the development of several lymphocytic responses in vitro. They demonstrated that resveratrol generally inhibits the proliferation of spleen cells induced by ConA, IL-2, or alloantigens, and more effectively inhibits the production of IL-2 and IFNγ by lymphocytes than the production of TNFα or IL-12 by macrophages [9]. Five years later, studies by Agrewala et al. showed that resveratrol and curcumin suppress the activity of T and B cells and macrophages, as proven by significant proliferation inhibition, antibody production, and lymphokine secretion. Curcumin was also found to impart immunosuppression mainly by down-regulating the expressions of CD28 and CD80 and up-regulating that of CTLA-4. Furthermore, resveratrol was found to function by decreasing the expressions of CD28 and CD80 and by augmenting the production of IL-10, but at the same time, had no effect on the percentage of CD4^+^CD25^+^ Treg cells [10]. Pan et al. demonstrated that resveratrol suppresses the protein kinase Cθ in peripheral blood T lymphocytes in a rat liver transplantation model [11]. Damo Xu et al. demonstrated that resveratrol could modulate murine collagen-induced arthritis by inhibiting Th17 and B-cell function [12]. However, Chul-Woo Kim et al. found that the administration of resveratrol suppresses the CD4^+^CD25^+^ cell population among CD4^+^ cells, down-regulates the secretion of TGF-β, and enhances IFNγ expression in CD8^+^ T cells both ex vivo and in vivo, leading to immune stimulation [13]. Thus, the effects of resveratrol on T cells and its specific molecular mechanisms are controversial.

Given that resveratrol has been proposed to account for the unique effects of red wine on lifespan and health and has been linked to physiological benefits, such as protection against cardiovascular disease [14,15], cancer, and age-related deterioration, a debate on the direct targets, downstream effectors, and molecular mechanism by which resveratrol improves health span has ensued. Silent mating type information regulation 2 homolog (Sirt1) is reported to be the direct target of resveratrol, or at least one of the downstream effectors of resveratrol [16,17]. Our previous study demonstrated that Sirt1 maintains periphery T cell tolerance, and that the ablation of Sirt1 leads to the enhancement of T cell activation and spontaneous autoimmune disease [18].

In the present study, we report for the first time that resveratrol inhibits T cell activation by enhancing the expression and deacytelase activity of Sirt1, thereby decreasing the acetylation of c-Jun, blocking the translocation of c-Jun into the nucleus, and finally inhibiting T cell activation. Animal experiments suggest that resveratrol could be used for the treatment of rheumatoid arthritis.

## Materials and Methods

### Mice

Six- to eight-week-old C57/BL6 mice and male DBA1 mice were purchased from Shanghai SLAC Laboratory Animal Co. Some of C57/BL6 mice were purchased from Jackson Laboratory. The Mir-34a transgenic mice used in this study were bred by Cyagen Bioscience, Inc. Sirt1^-/-^ mice were gifted by Dr. Michael W. McBurney. Mice were maintained in a barrier facility at Soochow University or Northwestern University. All animal experiments were approved by the Institutional Animal Care and Use Committees of Soochow University or Northwestern University.

### Antibodies

Anti-Sirt1 (B-10), anti-NFATC2 (4G6-G5), and anti-NFκb-P65 (C-20) antibodies were purchased from Santa Cruz Biotechnology, Inc. (Santa Cruz, CA). Anti-c-Jun (60A8) antibody was purchased from Cell Signaling Technology, Inc. (Danvers, MA). Anti-CD3 (145.11) and anti-CD28 (37.51) were purchased from Biolegend, Inc. (San Diego, CA).

### T cell proliferation assay

C57/BL6, FVB, or Mir-34a TG mice were sacrificed, their spleens were harvested, and red cells from the mice were lysed with ACK buffer. The CD4^+^ T cells were purified using anti-CD4 microbeads according to the manufacturer’s instructions (Miltenyi Biotec, Inc.). The purified CD4^+^ T cells were activated using plate-coated anti-CD3 antibodies with or without anti-CD28 antibodies (2.5 µg/ml) and with or without resveratrol (dissolved in DMSO, 0 µM to 25 µM). The same amount of DMSO was added to the negative control wells, some of which were labeled with CFSE, following the CellTrace™ CFSE cell proliferation kit manual (Molecular Probes-Eugene, Oregon) before being seeded onto the plates.

For the in vivo experiment, we evaluated T cell proliferation in the C57/BL6 mice immunized using bovine type II collagen emulsified with complete Freund’s adjuvant (CFA). Some of the mice were given resveratrol through daily intraperitoneal injection (IP) (25 mg/kg). On day 8 after immunization, a single-cell suspension of splenocytes was prepared and stimulated with collagen. The type II collagen used for T cell stimulation was denatured and then dissolved in 1 mg/ml warm PBS and centrifuged at 10,000 rpm for 3 min at room temperature. Afterward, 10 µl of supernatant was added to each well of the 96-well flat-bottomed plate well and cultured for 3 days. The cells were cultured using an RPMI1640 medium with 10% FCS. After 48 h of culturing, BrdU was added to the cell culture medium. Twenty-four hours later, the cells were fixed and DNA was denatured using FixDenat. The cells were then incubated with an anti-BrdU-POD antibody for 30 min, and then washed thrice with wash buffer. TMB substrate was added and the reaction product was quantified by measuring the absorbance (A370 nm to A492 nm) using a scanning multi-well spectrophotometer (ELISA reader). The CFSE-labeled cells were detected using FACS after culturing for 72 h to 96 h.

### Enzyme-linked immunosorbent assay for cytokine production (ELISA)

The cell cultured supernatant (treated with 0 µM or 25 µM of RES) was collected after 48 h and the serum was collected after administering RES or DMSO through 1 week of daily IP injections (25 mg/kg) on the C57/BL6 mice immunized with collagen. The mice were subjected to detect the production of cytokines, such as IL-2, IFNγ, IL-4, IL-5 and IgM, according to the protocol described in the eBioscience ELISA kit (San Diego, CA). The cell-cultured supernatant was diluted with a sample buffer and incubated in pre-coated with relative capture antibody for 1 hour at room temperature. After washed with wash buffer for three more times, the detection antibody was placed in the plate wells, incubated for another 1 h, and again washed thrice. Avidin–HRP was added to the wells and incubated for 30 min and washed five or six times with wash buffer. TMB substrate solution was added to each detection well and incubated for 15 min. The resulting reaction was stopped by adding 2N H_2_SO_4_ stop solution. The absorbance was then measured at 450 nm within 30 min of adding the stop solution.

### Immuno-precipitation and Western blot analysis

CD4^+^ T cells, both treated or not treated with resveratrol, were lysed with RIPA buffer containing 0.2 mM PMSF for 10 min. The supernatant was mixed with anti-c-Jun or anti-NFATC2 (4G6-G5) and anti-NFκb-P65 antibodies and then incubated on ice for 1 h. Afterward, 30 µl protein A beads (Roche Applied Science) were added to the mixture, which was shaken overnight at 4 °C. The supernatant was then discarded and the pellets were washed with RIPA buffer thrice. The 1×SDS loading buffer was added to the pellets. The mixture was boiled for 10 min and subjected to SDS–PAGE gel. The membrane was then blotted with c-Jun, NFATC2, and NFκb-P65 antibodies, with anti-acetylase and GAPDH as loading references.

### Confocal microscopy observation

The purified CD4^+^ T cells were either unstimulated or stimulated with anti-CD3 antibody plus anti-CD28 antibody. Part of the cells was treated with resveratrol for 24 h. These cells were cyto-spun onto slides and fixed. The expression of c-Jun was stained with anti-c-Jun antibody and anti-rabbit IgG-Cy5. The nuclear DNA was stained with DAPI and then observed under a Nikon A1 MP multiphoton imaging system.

### Induction and treatment of collagen-induced arthritis

Collagen-induced arthritis (CIA) was administered in DBA1 mice [19]. DBA1 mice were immunized by intra-dermal injection of 100 µg of bovine type II collagen (Chondrex Inc., Redmond, WA) emulsified in CFA. On day 21, the mice were boosted by intra-dermal injection of 100 µg of bovine type II collagen and incomplete Freund’s adjuvant (IFA). Resveratrol (Sigma, Gillingham, Dorset, UK) was dissolved in DMSO and administered intragastrically to the mice daily for eight weeks from the initial immunization. The control mice were fed the same amount of DMSO in PBS. The paw thicknesses of the mice were measured using a dial caliper. Two to three months later, the mice were sacrificed, their hind limbs were decalcified, and their joints sections were prepared and stained with hematoxylin and eosin.

### Statistical analysis

Statistical analysis was performed for all experiments using Student’s *t*-test. *P* values less than 0.05 were considered statistically significant.

## Results

### Resveratrol suppresses T cell activation in vitro

**Figure 1:**
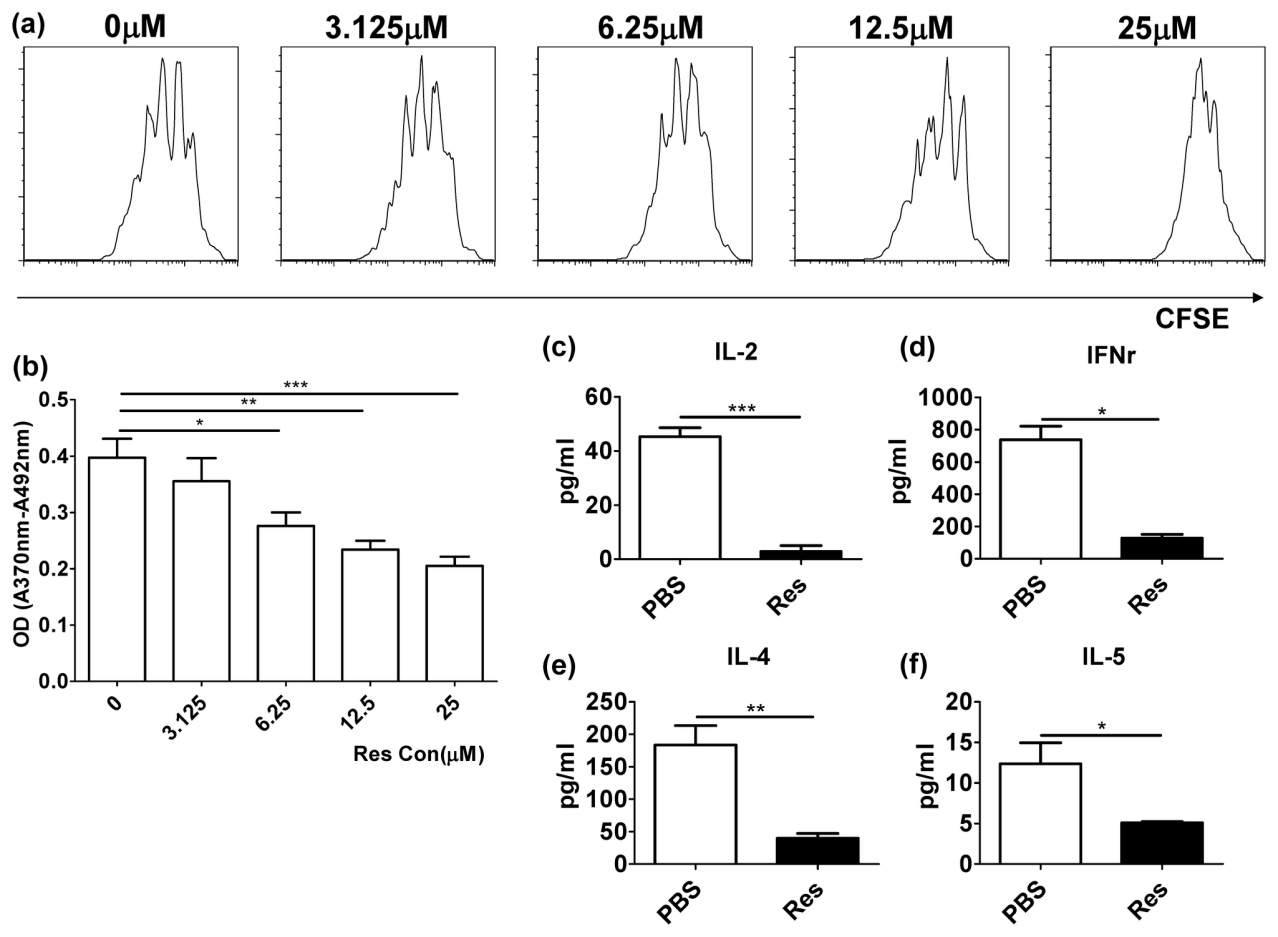
Resveratrol inhibited T cell activation in vitro and in vivo. (**a**) The proliferation of CD4^+^ T cells treated with different concentrations of resveratrol was evaluated by CFSE labeling assay. (**b**) The proliferation of CD4^+^ T cells treated with different concentrations of resveratrol was measured by BrdU proliferation assay. (**c**–**f**) The productions of cytokines in supernatant of cell cultures, including IL-2 (**c**), IFNγ (**d**), IL-4 (**e**) and IL-5 (**f**), were measured by ELISA (*P < 0.05, **P < 0.01, ***P < 0.001). The data are representative of at least five independent experiments.

First, we investigated whether resveratrol affects T cell activation. The CD4^+^ T cells were activated with anti-CD3/anti-CD28 antibodies and treated with different concentrations of resveratrol dissolved in DMSO, as shown in Figure 1. The CFSE (Figure 1a) and BrdU proliferation assays (Figure 1b) demonstrated that resveratrol inhibited T cell activation in a dose-dependent manner. ELISA showed that resveratrol inhibited both Th1 and Th2 cytokine productions, including IL-2 (P=0.0004), IFNγ (P=0.0022), IL-4 (P=0.095) and IL-5 (P=0.0484) (Figure 1c–f).

### Resveratrol suppresses T cell activation in vivo

**Figure 2:**
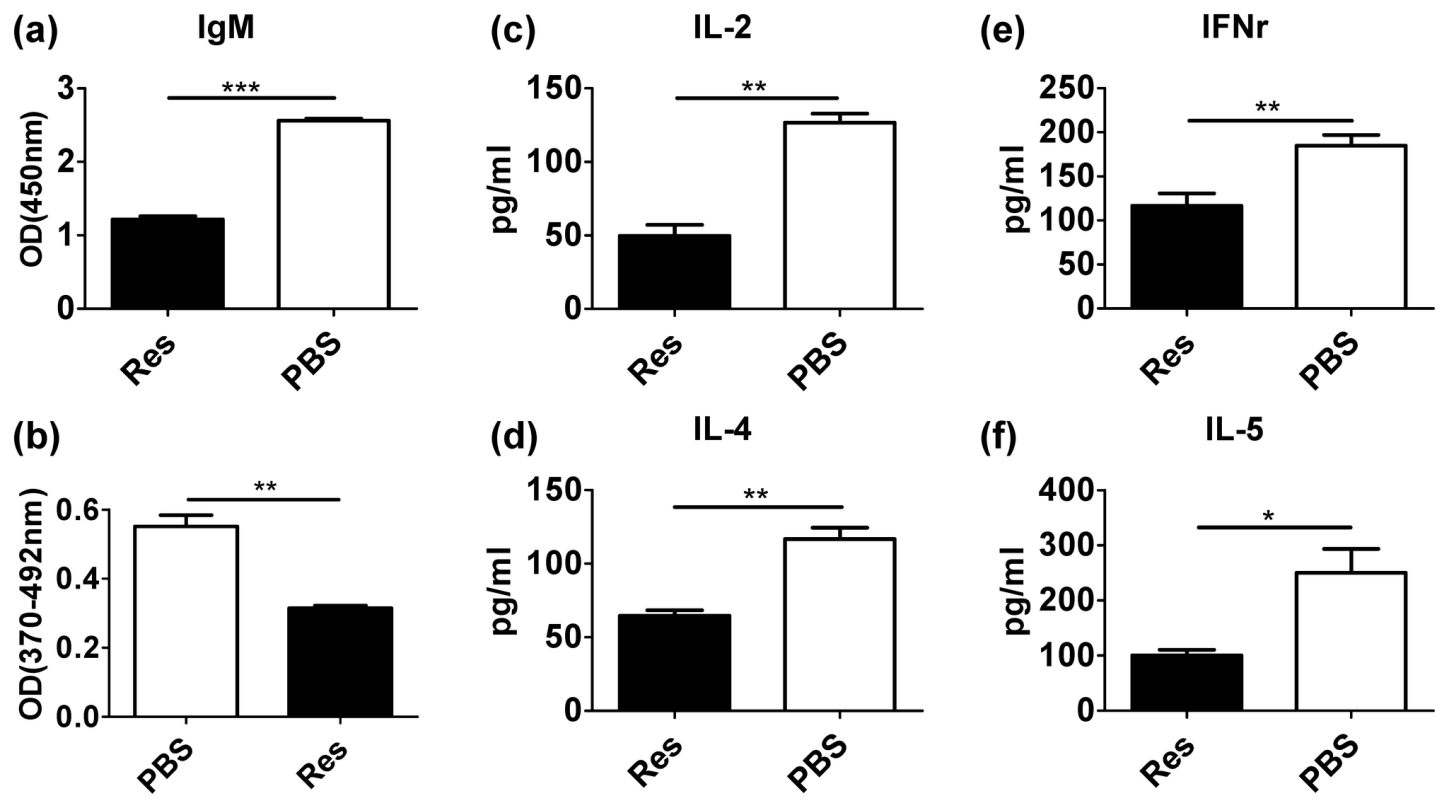
Resveratrol inhibited T cell activation and antigen-specific antibody production in vivo. (**a**) Collagen-specific IgM in the serum from immunized mice was detected by ELISA. (**b**) The proliferation of collagen-specific T cell prepared from collagen-immunized mice spleen were measured through BrdU proliferation assay. (**c**–**f**) The cytokines in the serum from mice immunized with collagen were detected by ELISA (*P < 0.05, ** *P* < 0.01, ***P < 0.001). The data shown are representative of three independent experiments.

To determine whether resveratrol effectively inhibits T cell activation in vivo, C57/BL6 mice were immunized with bovine type II collagen emulsified with complete Freund’s adjuvant. A number of mice were given resveratrol daily through IP (25 mg/kg). After 1 week, serum cytokines and collagen-specific IgM antibody were measured by ELISA. Single-cell suspensions of splenocytes were prepared and stimulated with collagen. The proliferation of splenocytes was evaluated by BrdU proliferation assay. The production of antigen-specific IgM was observed to be significantly lower in resveratrol-treated mice (P < 0.0001, Figure 2a). The antigen-specific T cell immune response was inhibited in resveratrol-treated mice unlike in non-treated mice (P = 0.0021, Figure 2b). Moreover, the production of cytokines, such as IL2 (P=0.0013), IFNγ (P=0.0098), IL-4 (P=0.0036) and IL-5 (P=0.0284), was significantly more inhibited in resveratrol-treated mice than in non-treated mice (Figure 2c–2f). These results suggest that resveratrol also inhibited T cell immune response and antibody production in vivo.

### Resveratrol inhibits T cell activation mediated by Sirt1

**Figure 3:**
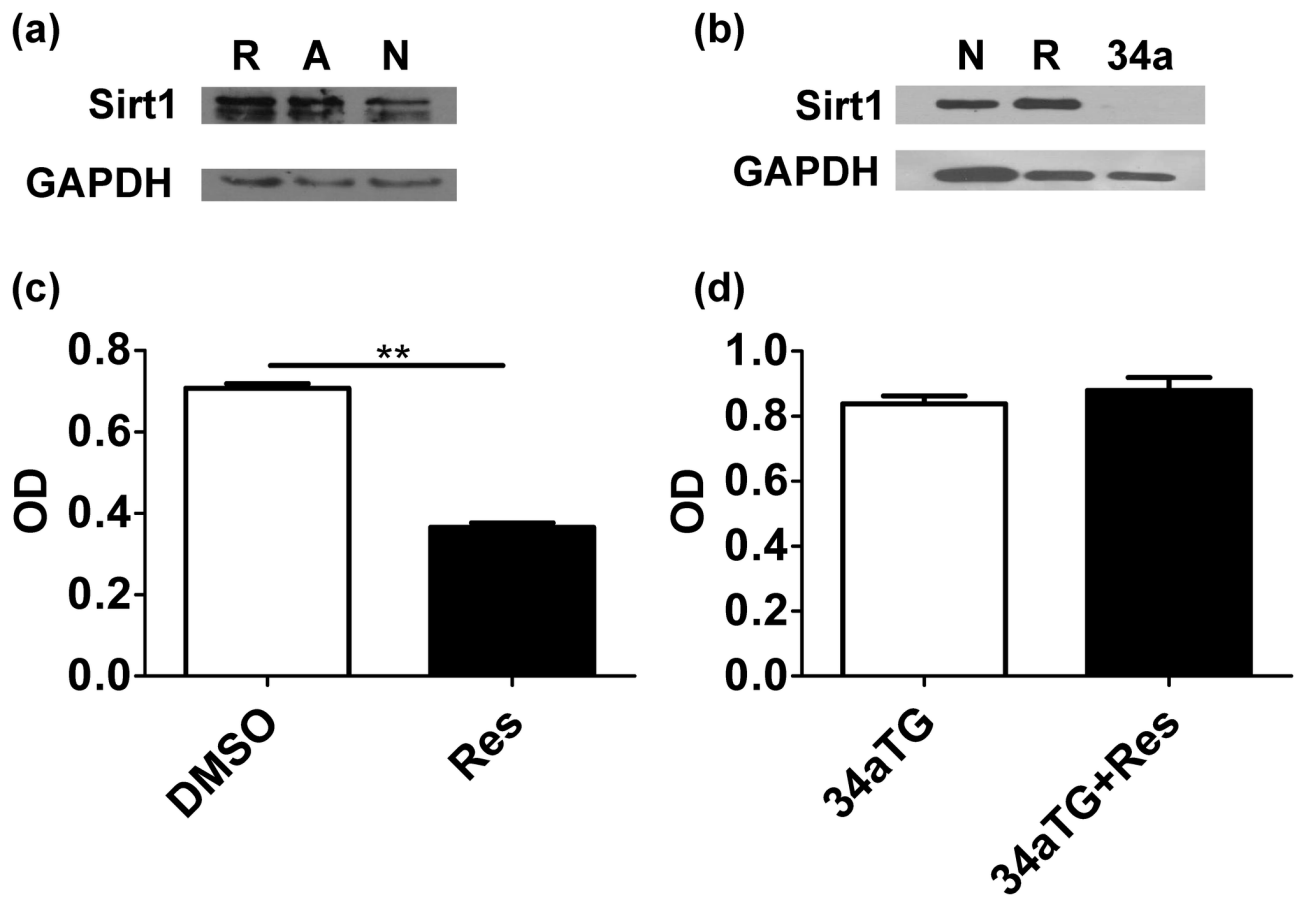
Resveratrol inhibited T cell activation by enhancing the expression of Sirt1. (**a**) The expression of Sirt1 in resveratrol treated (**R**), anti-CD3/CD28 antibody-activated (**A**), and naïve T cells (**N**) cells were detected through Western blot. GAPDH was used as loading reference. (**b**) The expression of Sirt1 in wild-type T cells (**N**), resveratrol-treated T cells (**R**), and mir-34a TG mouse T cells (**34a**). GAPDH was used as loading reference. (**c**, **d**) The proliferation of CD4^+^ T cells from wild-type mice and mir-34a TG mice, some of which were treated with resveratrol (**P < 0.01). The data are representative of three time-independent repetitions.

Given that Sirt1 is reportedly a potential target of resveratrol [15–17] and our previous study has demonstrated that Sirt1 maintained periphery T cell tolerance in mice [18], we investigated whether resveratrol inhibited T cell activation by enhancing the expression and activity of Sirt1. First, we treated naïve T cell with resveratrol. Western blot showed that the expression of Sirt1 in the naïve T cell was up-regulated after it was treated with resveratrol. Moreover, the expression of Sirt1 was more up-regulated in the activated T cells compared with that in the naïve T cells (Figure 3a, 3b). To further investigate whether resveratrol affects T cells through Sirt1, we constructed mir-34a-over-expressed plasmids (pMDH-PGK-GFP-mir34a) and created the mir-34a transgenic mice (unpublished data). Given that Sirt1 is one of the targets of mir-34a [20], we used these mice as Sirt1 knock-down mice. Western blot showed that Sirt1 was undetectable in mir-34a-TG mice (Figure 3b). Although resveratrol could inhibit the activation of wild-type T cells (P = 0.0022, Figure 3c)), it did not affect the T cells from mir-34aTG mice (P=0.5129, Figure 3d), suggesting that resveratrol only inhibited T cell activation by enhancing the expression of Sirt1 in the T cells.

### Resveratrol enhances Sirt1 deacetylase activity on c-Jun

**Figure 4:**
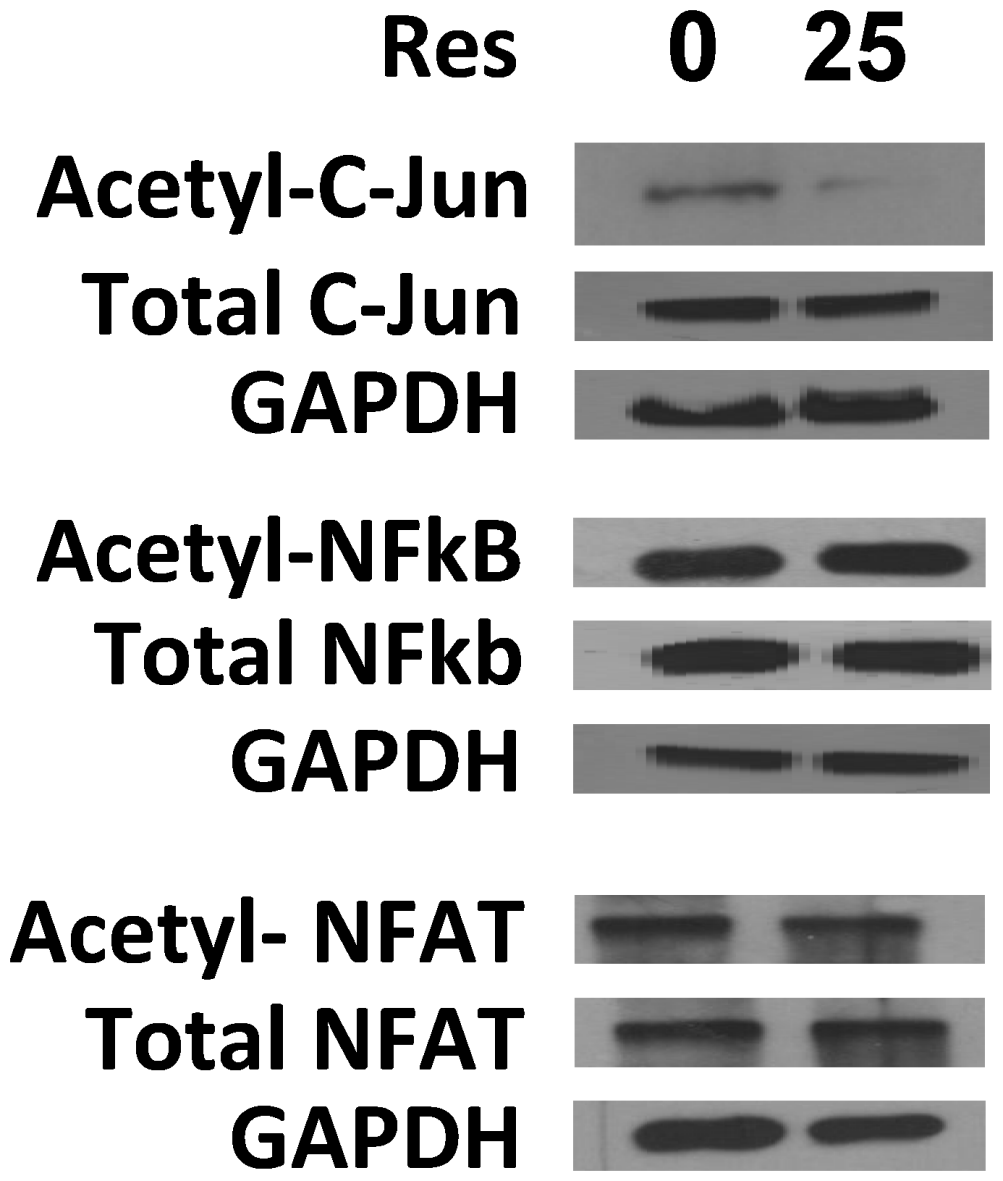
Resveratrol enhanced the acetylase activity of Sirt1 on c-Jun. The cell lysis of activated T cells was precipitated with antibodies against c-Jun, NFκB, or NFAT, blotted with anti-acetylase antibody, and then blotted with antibodies against C-Jun, NFκB, NFAT, and GAPDH as references. The pictures are representative of three more repetitions.

**Figure 5:**
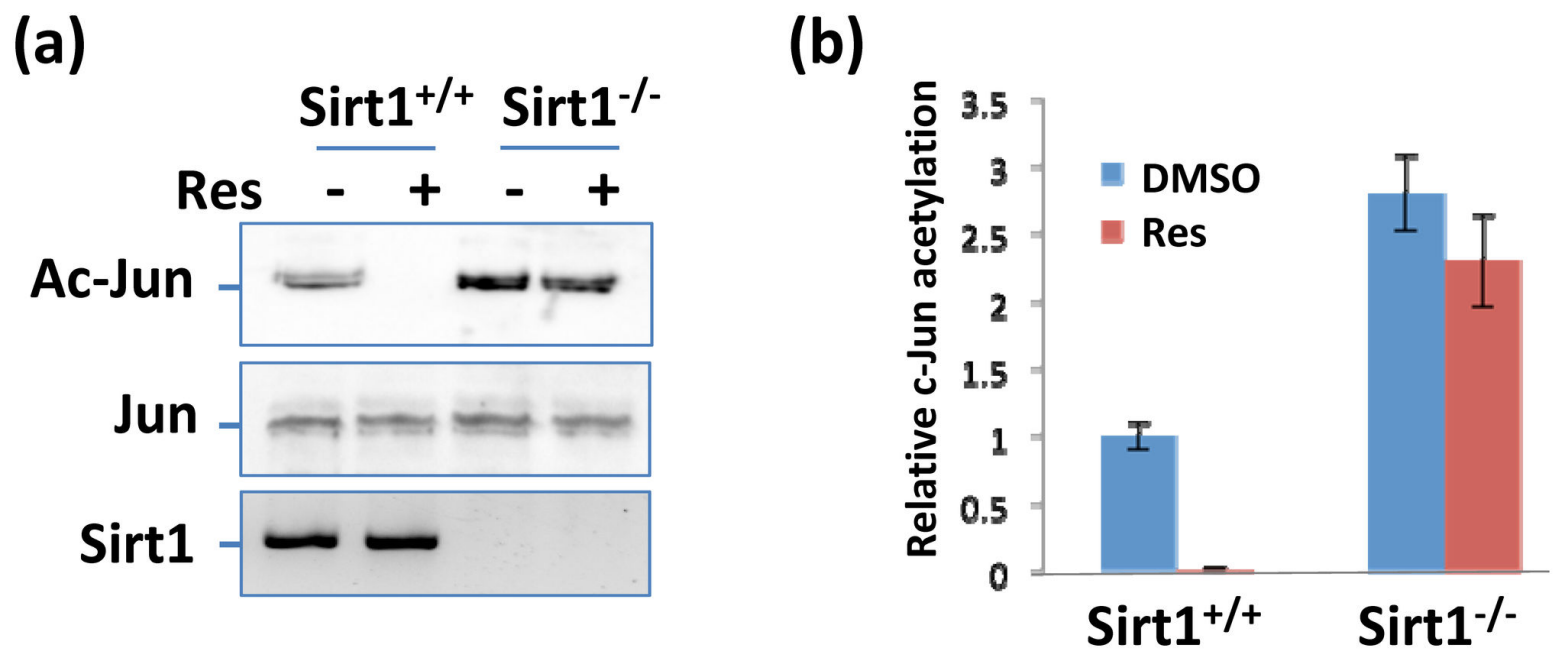
Primary T cells were isolated from WT and Sirt1^-/-^ mice and cultivated with anti-CD3 (5 µg/ml) and anti-CD28 (2 µg/ml) for overnight. Cells were then treated with Res (10µg/ml) for additional 8 hours. (**a**) c-Jun acetylation in the lysates of treated cells was determined by immunoprecipitation with anti-c-Jun antibody and western blotting with anti-acetyl-lysine Abs (top panel). The expression levels of c-Jun (middle panel) and Sirt1 (bottom panel) in the whole cell lysate were confirmed by western blotting. (**b**) The levels of acetylated c-Jun were quantified and normalized with total c-Jun and its relative levels are shown. Error bars represent data from three independent experiments (mean + SD).

Sirt1 is a deacetylase. To determine whether resveratrol affects the deacetylase activity of Sirt1, the T cells were treated with resveratrol and activated with anti-CD3/anti-CD28 antibodies. c-Jun, NFAT, and NFκb proteins were immuno-precipitated with anti-c-Jun, anti-NFAT, and anti-NFκb antibodies, respectively. By blotting the precipitated protein with anti-acetylated lysine, the acetylations of NFAT and NFκb did not clearly change although the acetylation of c-Jun was decreased in resveratrol-treated T cells (Figure 4). To further verify that acetylation decreasing of c-Jun induced by reseveratrol is dependent on Sirt1, we treated T cells isolated from Sirt1^+/+^ and Sirt1^-/-^ mice with resveratrol, and c-Jun were precipitated with anti-c-Jun antibody, then blotted with anti-acetylation antibody and anti-c-Jun antibody, we found that acetylation of c-Jun was significantly decreased in Sirt1^+/+^ T cells treated with resveratrol, compared with that in non-treated wild type T cells. but similar amount of acetylated c-Jun were observed between non-treated and resveratrol-treated Sirt1^-/-^ T cells, meanwhile, the amount of total c-Jun were similar (Figure 5). These all results further suggest that resveratrol inhibits T cell activation by decreasing the acetylation of c-Jun, and this is totally dependent on sirt1 existing.

### Resveratrol inhibits the translocation of c-Jun into the nucleus upon T cell activation

**Figure 6:**
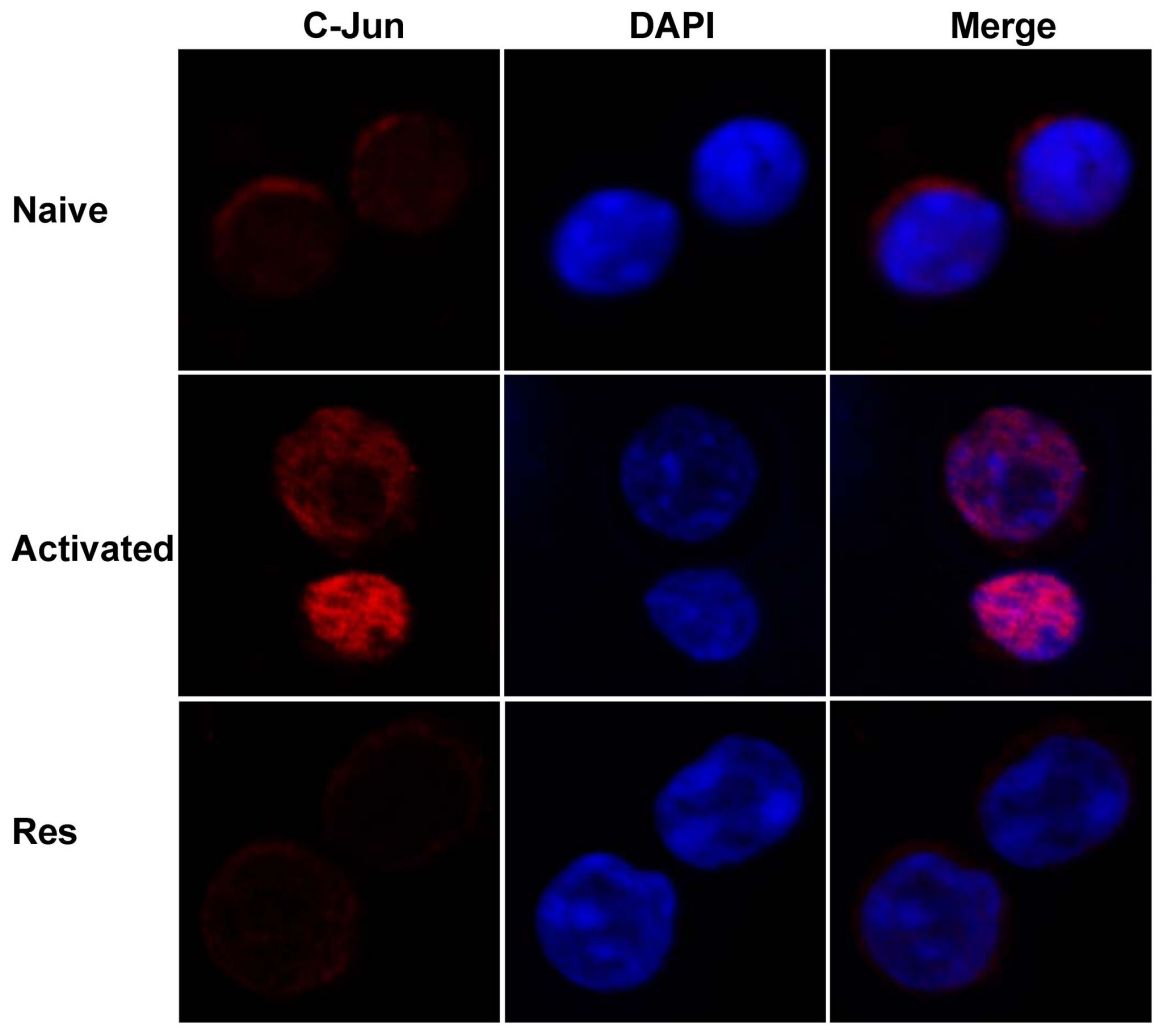
Resveratrol blocked the translocation of c-Jun into the nucleus. Naïve CD4^+^ T cells, anti-CD3/CD28 antibody-activated CD4^+^ T cells, and resveratrol-treated CD4^+^ T cells were stained with Cy5-labeled anti-c-Jun antibody and DAPI, and then observed under a confocal microscope. Experiments were repeated more than four times.

Upon T cell activation, c-Jun is translocated from the plasma to the nucleus. To further study the mechanisms of resveratrol during T cell activation, we investigated whether changing the acetylation of c-Jun affects the translocation of c-Jun upon T cell activation. To verify this hypothesis, purified CD4^+^ T cell were treated with resveratrol, activated with anti-CD3/CD28 antibodies, and then stained with anti-c-Jun antibody. The samples were mounted with DAPI, and then the slides were sealed. Through confocal microscopy, we found that in naïve T cells, c-Jun was distributed in the plasma around the nucleus. Once the T cells were activated, c-Jun moved into the nucleus. However, in the resveratrol-treated T cells, most portions of the c-Jun were arrested in the plasma (Figure 6). These results suggest that resveratrol inhibits the translocation of c-Jun into the nucleus upon T cell activation.

### Treatment with resveratrol reduces the incidence and severity of collagen-induced rheumatoid arthritis

**Figure 7:**
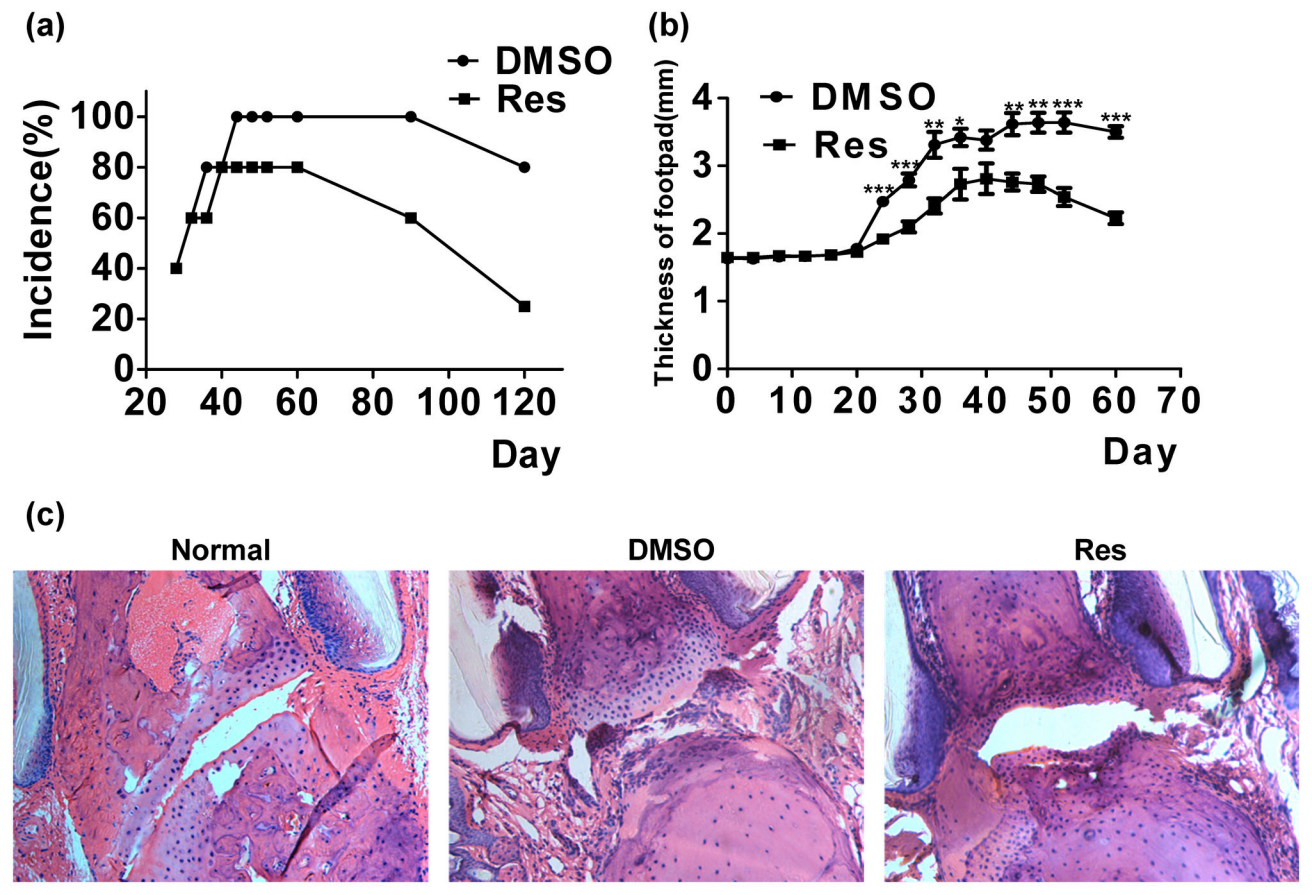
Resveratrol reduced the incidence and severity of CIA. (**a**) The incidence of CIA in resveratrol-treated DBA1 mice was significantly lower compared to that of untreated CIA DBA1 mice. (**b**) The thickness of footpad in resveratrol-treated and untreated CIA DBA1. (**c**) CIA mouse joint sections were stained with H&E (*P < 0.05, **P < 0.01, ***P < 0.001). Each group had six mice. The experiments were repeated three times.

To evaluate whether resveratrol can prevent the development of CIA, DBA1 mice were induced with CIA on day 0 and administered daily with resveratrol (20 mg/kg per mouse/day) in an intra-gastric manner from day 0 for eight consecutive weeks. The development of arthritis was monitored until day 60. Control mice displayed severe CIA. As observed from day 24, resveratrol-treated mice showed significantly reduced disease incidence (Figure 7a) and footpad thickness (Figure 7b). Histological analysis demonstrated that infiltrated cells in the joint, synovial hyperplasia, and adjacent cartilage, as well as bone erosion, were clearly reduced in the resveratrol-treated mice unlike in the control mice (Figure 7c). These results suggest that resveratrol can prevent the development of CIA.

## Discussion

In 1992, Siemann and colleagues posited that resveratrol in red wine has cardioprotective effects [21]. Numerous studies have also shown that resveratrol can prevent or slow the progression of various conditions, including cancers, cardiovascular diseases, and ischemic injuries, as well as enhance stress resistance and extend lifespan [1–9]. Attempts to demonstrate favorable effects in vitro have met with almost universal success and have led to the identification of multiple direct targets of resveratrol, such as COX, PPAR, eNOS and Sirt1 [22–24]. Park et al. provided a detailed picture of the mechanism through which resveratrol activates AMPK and Sirt1 to produce the following metabolic benefits. Resveratrol inhibits PDEs, leading to increased cAMP levels, Epac1 activation, elevated intracellular calcium, and AMPK activation. Calorie restriction and other behavioral inputs also activate AMPK. In the downstream activation of AMPK, an increase in NAD^+^ levels leads to Sirt1 activation, which promotes beneficial metabolic changes primarily through deacetylation and activation of PGC-1α. In a parallel pathway, increased cAMP levels activate PKA, which directly phosphorylates and activates Sirt1 [14,15].

For the immune system, a few studies have shown that resveratrol participates in the activation of T cell, B cell, and macrophage, and is involved in CD4^+^CD25^+^ regulatory T cell suppressive functions [10,11,13]. However, some of these studies are controversial, and the mechanisms of resveratrol in relation to T cell activity remain unclear. Sirt1 is believed to be a receptor of resveratrol, at least, one of downstream signal pathway [14–17]. Our previous study has demonstrated that Sirt1 is involved in periphery T cell tolerance; ablation of Sirt1 in T cells could induce hyper-activation of T cells and lead to spontaneous autoimmune disease [18]. Thus, we deduced that resveratrol may affect T cell activation through Sirt1. To confirm this hypothesis, T cells were treated with resveratrol and activated with anti-CD3/CD28 antibodies in vitro. BrdU staining and CFSE labeling showed that T cell activation was inhibited in the resveratrol-treated group unlike in the non-treated group. Moreover, the cytokine productions in resveratrol-treated T cells, such as Th1 type cytokines and Th2 type cytokines, decreased in a dose-dependent manner. Resveratrol could also inhibit T cell activation and production of antigen-specific antibody in vivo. Our subsequent experiments showed that Sirt1 expression was higher in resveratrol-treated T cells than in naïve T cells. Sirt1 was also up-regulated in activated T cells. The inhibitory effect of resveratrol on T cell activation disappeared in Sirt1 knockdown T cells. These results suggest that the inhibition of T cell activation by resveratrol was mediated by Sirt1. Sirt1 is an important deacetylase involved in numerous molecular events, including metabolism [25–28], cancer [29,30], embryonic development [31,32] and immune tolerance [33,34]. To comprehensively explain the mechanisms of resveratrol involved in T cell activation, we examined the Sirt1 deacetylase activity on c-Jun, NFκb, and NFAT, as well as the pivotal transcriptional factors in T cell activation [35–37]. Western blot showed that resveratrol increased Sirt1 acetylase activity on c-Jun, but not on NFAT and NFκb in T cells. However, resveratrol cannot decrease the acetylation of c-Jun in Sirt1^-/-^ T cells, strongly suggests that acetylation change of c-Jun is totally dependent on Sirt1. Once T cells were activated, c-Jun was translocated into the nucleus. However, this action of c-Jun was blocked in resveratrol-treated T cells. Thus, resveratrol can clearly inhibit T cell activation by increasing the expression of Sirt1 and the deacetylase activity of Sirt1 on c-Jun, which impedes the translocation of c-Jun into the nucleus.

Abnormal T cell activation is generally involved in many pathogeneses, such as rheumatoid arthritis, systemic lupus erythematosus and experimental allergic encephalomyelitis [38–41]. Given that resveratrol can inhibit T cell activation and reduce cytokine production, whether resveratrol can prevent autoimmune disease progression remains unknown. To address this concern, we treated mice with resveratrol with RA induced by collagen. The resveratrol-treated mice showed significantly reduced disease incidence and footpad thickness. Histological analysis demonstrated that infiltrated cells in the joint were clearly reduced in the resveratrol-treated mice compared with the control mice. These results suggest that resveratrol can prevent the development of CIA. Given that resveratrol is a natural component of grapes, this study confirms that drinking wine regularly and eating more red grapes are beneficial for preventing autoimmune disease occurrence. Resveratrol can also be used in clinics for treating inflammation induced by T cell activation, as well as for treating other T cell-related diseases. But some concerns are still necessary.

Regarding the Th17 cells and Treg play very important roles in autoimmune disease, Damo Xu group showed the arthritis-protective effects of resveratrol were associated with reduced numbers of Th17 cells and the production of IL-17 in DLN [12]. For EAE model, Thomas M. Petro group showed that resveratrol protection against EAE is not associated with declines in IL-17^+^ T cells but is associated with rises in IL-17 ^+^ /IL-10^+^ T cells and CD4^-^IFN-γ^+^ and with repressed macrophage IL-6 and IL-12/23 p40 expression [42]. Interestingly, the function of resveratrol on Treg cells seems to be benefit on T cell activation. CD4^+^CD25^+^Foxp3^+^ cells were significantly reduced in the total splenocytes as well as tumor tissues from HS-1793 (resveratrol analog)-administered mice, and the production of TGF-β inducing Treg showed a similar pattern [43]. How does resveratrol function on T cell activation in bidirectional way? For autoimmune disease model it seems to exert inhibitory function in ours and others studies, but for tumor model, resveratrol seems to reduce the suppressive function of Treg so that inhibits the tumor growth. What induces this difference in different animal model? We guess the final effective manifestation of resveratrol may be dependent on two hits, one hit is resveratrol itself, but another hit is provided by specific microenvironment under different disease model. Anyway, further experimental evidence is urgent to verify this hypothesis and resolve these disputes.
